# Medical professionalism education: a systematic review of interventions, outcomes, and sustainability

**DOI:** 10.3389/fmed.2025.1522411

**Published:** 2025-03-03

**Authors:** Asil Sadeq, Shaista S. Guraya, Brian Fahey, Eric Clarke, Abdelsalam Bensaaud, Frank Doyle, Grainne P. Kearney, Fionnuala Gough, Mark Harbinson, Salman Yousuf Guraya, Denis W. Harkin

**Affiliations:** ^1^Centre for Professionalism in Medicine and Health Sciences, Faculty of Medicine and Health Sciences, RCSI University of Medicine and Health Sciences, Dublin, Ireland; ^2^Institute of Learning, Mohammad Bin Rashid University, Dubai, United Arab Emirates; ^3^Department of Health Psychology, School of Population Health Sciences, RCSI University of Medicine and Health Sciences, Dublin, Ireland; ^4^School of Medicine, Dentistry and Biomedical Sciences, Queens University Belfast, Belfast, United Kingdom; ^5^Clinical Sciences Department, College of Medicine, University of Sharjah, Sharjah, United Arab Emirates

**Keywords:** medical professionalism, medical professionalism education, professional behaviour, systematic review, sustainability, professional identity formation

## Abstract

**Introduction:**

Medical professionalism (MP) is a vital competency in undergraduate medical students as it enhances the quality and safety of patient care as it includes professional values, attitudes and professional behaviours (PB). However, medical institutes are uncertain about how optimally it can be learnt and assessed. This review aims to systematically provide a summary of evidence from systematic reviews reporting MP educational interventions, their outcomes and sustainability to foster PB.

**Methods:**

Eight major databases (CINAHL, EMBASE, ERIC, Health business, Medline, OVID, PsycINFO, SCOPUS and Web of Science) and grey literature were systematically searched from database inception to June 2024. The inclusion criteria were (1) systematic review studies (2) of educational interventions of any type; (3) targeting any aspect of MP; (4) provided to undergraduate medical students; and (5) with no restrictions on comparator group or outcomes assessed. A qualitative narrative summary of included reviews was conducted as all included reviews did not conduct quantitative nor meta-analysis of results but rather a qualitative summary. Methodological quality of included reviews was assessed using A MeaSurement Tool to Assess systematic Reviews (AMSTAR) 2 tool.

**Results:**

The search identified 397 references for eligibility screening. Ultimately, eight systematic reviews were deemed eligible for inclusion. The majority of these reviews have reported a successful improvement in various aspects of MP (i.e., MP as a whole, empathy and compassion) through teaching and exposure to hidden curriculum. The included studies displayed significant methodological heterogeneity, with varying study designs and assessment methodologies to professional outcomes. A gap remains in reporting the sustainable effect on professionalism traits and on a standardised approach to MP teaching.

**Conclusion:**

This review suggests that more interventions are needed in this area with a focus on methodological quality and teaching methods in a multicultural context to support PB and professional identity formation.

**Clinical trial registration:**

PROSPERO [CRD42024495689].

## 1 Introduction

Medical professionalism (MP) is defined as encompassing a range of values, professional behaviours (PB), and attitudes that are expected from healthcare care professionals to maintain public trust and ensure patient safety (1). The evolution of MP towards a more patient-centred approach has significantly escalated over time, particularly in the late 20th century (2–5). Today, MP encompasses a range of attributes, including compassion, integrity, accountability, and a commitment to continuous learning (1). MP is critical for maintaining clinical competence and ensuring that healthcare decisions prioritize patient welfare above all other considerations (6). As modern healthcare becomes increasingly complex, driven by technological advances and ethical challenges, maintaining high standards of MP remains essential for promoting equitable, compassionate, and patient-centred care (3).

Within medical education, MP is a core component as highlights the importance of cultivating professionalism at three distinct levels: individual (i.e., empathy, decision making, and accountability); institutional (i.e., commitment to integrating professionalism into clinical placement); and societal level (i.e., patient care and public trust in the healthcare systems) (7). In recent years, medical schools strive to formalize and standardize this aspect of the curriculum provided to undergraduate medical students (UMS) (7). Traditionally, MP was learned implicitly through role modelling and clinical exposure at a postgraduate level (8). However, recent educational approaches advocate for explicit teaching, assessment, and reflection on professionalism to better prepare students for the complex professional dilemmas and challenges of clinical practice, these can include individual burnouts, cultural resistance/systematic pressure and societal signification of patient-centred care (9–12). This shift requires continuous improvement of curricula, including the integration of feedback mechanisms that allow students to reflect on and enhance their ethical conduct over time. Nevertheless, challenges persist, particularly due to the variability in how professionalism is defined across institutions and cultural contexts. This inconsistency creates obstacles to developing standardized curricula and objective assessment tools (10). Moreover, there is no clear consensus on which educational strategies are most effective for MP, especially in diverse, multicultural environments (13).

While these challenges remain, recent systematic reviews underscore the effectiveness of multifaceted interventions that blend theoretical knowledge (i.e., theory of constructivism, theory of planned behaviour, and social learning theory) with practical experiences (i.e., experiential learning) while also recognizing the critical influence of the learning environment on the development of PB and professional identity formation (14, 15). Despite these promising interventions, the literature remains limited regarding the long-term sustainability of these efforts aimed at fostering professionalism (16, 17). Many existing studies lack rigorous methodological designs and comprehensive evaluations of long-term outcomes (2). This lack of longitudinal data raises concerns about whether early gains in professionalism are sustained throughout the clinical years of medical training, where PB are particularly critical.

Educational interventions and efforts help ensure that MP teaching is not only relevant but also impactful (18, 19). However, to fully optimize these efforts, it is crucial to understand which approaches are most effective and sustainable. Thus, this review seeks to address the existing gaps by systematically evaluating the effectiveness of various professionalism education interventions. In particular, it will assess their impact on teaching methods, PB development, and the long-term sustainability of these interventions among medical students. By doing so, the review may provide clearer guidance on which methods lead to enduring improvements in MP.

## 2 Method

This systematic review, also known as an umbrella review (systematic review of systematic reviews) was conducted to systematically summarize the published systematic reviews in this area (20). The reporting was in accordance with the Preferred Reporting Items for Systematic Reviews and Meta-Analyses (PRISMA) statement (21). The protocol of this systematic review was prepared using PRIMSA-Protocol statement and registered on PROSPERO [CRD42024495689]. The PICO framework guiding this review is as follows:

- Population: Undergraduate medical students (preclinical and clinical).
- Interventions: Educational interventions designed to foster at least one attribute of MP (e.g., reflective practice, peer feedback, portfolios).
- Comparisons: Studies with and without comparator.
- Outcomes: all reported outcomes were included.

### 2.1 Search strategy

Eight major data bases were systematically searched (CINAHL, EMBASE, ERIC, Health business, Medline OVID, PsycINFO, SCOPUS and Web of Science) and grey literature from inception of data base to June 2024 to identify relevant systematic reviews. The search terms included the keywords *“Medical professionalism” “humanism” “Professional behaviour” “undergraduate medical students” “educational interventions”* and their appropriate synonyms to the title/abstract/keywords fields. The search was restricted to English language and where applicable, the search was either filtered to *“systematic review”* methodology or this was added to the search string. The search strategy was developed with the information specialist from the library in the Royal College of Surgeons in Ireland.

### 2.2 Study selection

All retrieved references were exported to Endnote 20 ®; duplicates were removed, and then imported into COVIDENCE.org, where duplicates were automatically repeated. Two reviewers independently screened references for title and abstract then for full text screening. Any conflicts between the two reviewers were resolved through discussion with a third reviewer. The same strategy was conducted for data extraction and quality assessment.

### 2.3 Data extraction and quality assessment

A data extraction form was developed with a team of expert researchers in systematic reviews and the topic of MP. The data extracted include characteristics of (i) systematic review (i.e., first author, publication year, aim, search date and number and type of study included); (ii) interventions included (type, duration, frequency, follow up, mode of delivery, themes of MP targeted); (iii) participants (number, undergraduate level/year, comparator characteristics); (iv) outcomes assessed; (v) key finding; and (vi) conclusion and suggestions.

The methodological quality of systematic reviews included was assessed using the A MeaSurement Tool to Assess systematic Reviews (AMSTAR) 2 tool (22) which includes a checklist of 16 criteria that critically appraises if the study had followed a comprehensive systematic protocol, bias and validity of conclusions. Two independent reviewers provide *“yes,” “no,”* or *“partial Yes”* voting for each of the 16 criteria. The final AMSTAR 2 scoring was automatically categorised as critically low, low, moderate and high quality using the online ASMTAR calculator available online on https://amstar.ca/Amstar_Checklist.php.

### 2.4 Data analysis

A narrative summary of included systematic review was conducted s as all included reviews reported qualitative findings rather than a statistical analysis. Thus, meta-analysis of included reviews was not applicable.

## 3 Results

**Figure 1:**
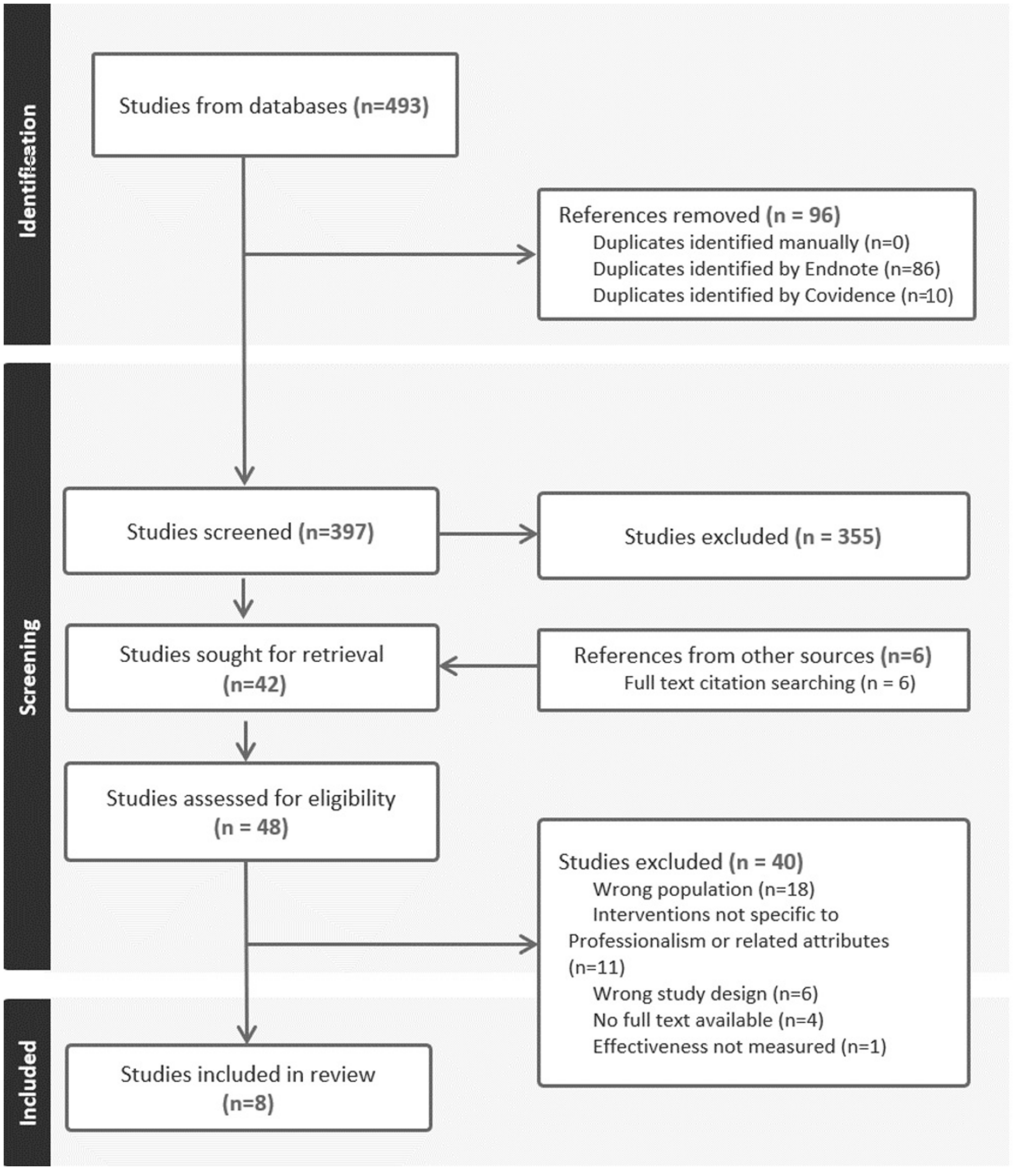
Preferred reporting items for systematic reviews and meta-analyses (PRISMA) flowchart (21).

The search identified 397 references for title and abstract screening after the removal of duplicated (*n* = 96) (Figure 1). A total w48 full text studies were screened for eligibility, and 40 references did not meet the inclusion criteria. Ultimately, eight systematic reviews were eligible for inclusion (23–30).

### 3.1 Characteristics of relevant reviews

The eight included systematic review were published between 2011 and 2023 with an overall search duration from the inception of databases until 2022. These systematic reviews included a total of 367 studies. A total of 117,875 UMS at both preclinical and clinical level of education are included in the retrieved studies, except for one study which targeted only students during the preclinical stage only (25). The included studies displayed significant methodological heterogeneity, with varying study designs (e.g., qualitative, quantitative, and mixed-method approaches) and differing methodologies for assessing outcomes. A detailed description of included studies and participants is provided in Table 1.

**Table 1:** Characteristics of relevant reviews.

Author, year	Aim	Medical professionalism	Year	Studies (*N*)	Participants (*N*) + Education level
Franco et al., 2016 (23)	“To review the characteristics of portfolios and their outcomes for teaching professionalism to undergraduate medical students”	Medical professionalism as a whole	Inception—2015	11 studies Qualitative (*n* = 8) Quantitative (*n* = 2) Mixed methods (*n* = 1)	1,326; preclinical and clinical students
Guraya et al., 2016 (24)	“To identify effective teaching strategies for medical professionalism”	Medical professionalism as a whole	2005–2015	48 studies Qualitative and quantitative report, and empirical studies	Not speified; preclinical and clinical students
Ghosh et al., 2018 (25)	“To highlight practices adopted by medical schools that enhance the implementation of the “hidden curriculum” in human dissection, fostering professionalism among students”	Empathy and compassion	Not specified. Included studies were published from 1994–2017	Not specified	Not specified; preclinical students
Lerchenfeldt et al., 2019 (26)	“To examine the utilization, effectiveness, and quality of peer feedback during collaborative learning in medical education.”	Medical professionalism as a whole	1997–2017	31 studies Quantitative (*n* = 15) Qualitative (*n* = 3) Mixed methods (*n* = 13)	±4,849; preclinical and clinical students
Rattani et al., 2021 (29)	“To evaluate and assess the functional use and application of short form audiovisual didactic supplements or “icebreakers” in medical ethics and professionalism teaching”	Medical professionalism as a whole	Inception—2019	13 studies Commentaires (*n* = 10) Qualitative (*n* = 2) Quantitative (*n* = 1)	Not specified; preclinical and clinical students
Menezes et al., 2021 (28)	“The review is associations between spectrum effectiveness, frequency of teaching and outcomes on empathy and compassion”	Empathy and Compassion	2015–2020	24 Studies Randomised Controlled Trials (*n* = 12) Controlled trials (*n* = 4) Single group pre/post test (*n* = 6) Single group post test (*n* = 2)	2,657; preclinical and clinical students
Wang et al., 2022 (30)	“To systematically review and synthesize studies investigating the predictors of compassion and related constructs (such as empathy) among medical students”	Empathy and compassion	Inception-2020	222 Empirical studies Quantitative (83%) Qualitative (12%) Mixed methods (4.2%)	108, 112; preclinical and clinical students
Leung et al., 2023 (27)	“To review the literature regarding the role of reflective practice in fostering empathy, wellbeing and professionalism in medical students”	Medical professionalism as a whole + Empathy and compassion	2010–2022	18 studies Qualitative studies (*n* = 9) Quantitative studies (*n* = 4) Mixed methods (*n* = 5)	931; preclinical and clinical students

### 3.2 Characteristics of interventions within relevant reviews

As described in Table 2, included studies involved education interventions that (i) were either provided either in-person (25, 26, 29) or using both in-person and an online platform (23, 24, 27, 28) (ii) were either embedded within the curriculum or provided as a separate session/workshop, as reported by seven SRs (23–29); (iii) ranged from single sessions to multiple sessions, as reported by five references (23, 25, 27–29); (iv) had a duration from 0.5 to 150 min, as reported by two references (28, 29); and (v) either compared with standard teaching, did not have a comparator group, or compared two teaching methods, as reported by five references (26–30). There was considerable heterogeneity in the interventions used across the studies, which ranged from reflective practices to peer feedback and audiovisual tools. This variability limits the direct comparability of results.

**Table 2:** Characteristics of interventions included in relevant reviews.

Author, year	Intervention type + MOD	Duration and frequency of interventions	Follow up	Comparator
Franco et al., 2016 (23)	Use of portfolios for teaching and assessing professionalism; included learning diaries and evidence of assessment. MOD: Electronic portfolios (web-based, email, software) + Paper-based portfolios	Ranging from weekly, monthly, regular or less frequent submissions. Most portfolios were used for one academic year or more.	Reflection and feedback were emphasized as critical components for the successful long-term development of MP.	Not specified
Guraya et al., 2016 (24)	Reflective practices-based studies; interactive lectures, vignettes, small group teaching, simulations, video reviews, experiential learning, dependent and independent learning, curriculum integration, early clinical exposure and faculty development programmes. MOD: Virtual or in-person.	Not specified	Not specified	Not specified
Ghosh et al., 2018 (25)	Showing video clips of donor interviews, interacting with family members of donors, and organizing memorial services. MOD: Virtual and/or in-person	Ranging from one-time event to ongoing throughout the anatomy dissection course and regular interactions through emails and letters.	Not specified, but suggests that the impact of these interventions can be long-lasting, shape students’ attitude, and behaviour throughout the medical careers and may help students maintain an empathetic perspective throughout their professional training.	Not specified
Lerchenfeldt et al., 2019 (26)	Problem-based learning and team-based learning. MOD: In-person	Not specified—embedded within curriculum	The long-term impact was discussed in some studies, especially in terms of professional development.	Included studies often did not describe comparator characteristics clearly.
Rattani et al., 2021 (29)	The use of short form audiovisual media in the form of trigger films or short films/videos + the use of clips from TV and films MOD: not specified	Not specified; audio-visual content duration ranged from 0.5 to 150 min	Not specified	Comparisons were drawn between different types of audio-visual media (trigger films, short films, TV clips).
Menezes et al., 2021 (28)	Multiple teaching modalities including virtual hangouts, online surveys, computerised tasks, didactic, small group discussion, simulations, service-learning experience and early clinical exposure MOD: Virtual and in-person	Integrated within curriculum; Ranging from single session (1–2 h) to multiple sessions throughout academic year(s)	Some studies followed up after a period of time and showed some improvement in empathy and compassion	Standard curricular activity (teaching of empathy and compassion)
Wang et al., 2022 (30)	No specific interventions. The study focused on predictors of compassion and empathy, including personal factors, environmental factors, patient/family factors, and clinical factors; it employed self-report questionnaires, interviews, focus groups, and various measurement scales such as the JSPE and IRI	N/A	The review emphasized the need for more research into long-term impacts, particularly regarding environmental and clinical factors.	Studies included a wide range of factors as predictors, including sociodemographic factors, training-related factors, dispositional characteristics, and quality of life indicators.
Leung et al., 2023 (27)	Reflective practice methods (Balint groups, reflective writing and some direct teaching) MOD: Virtual and in-person group sessions	Ranging from single sessions to weekly/ biweekly sessions.	Not specified	Some did not receive and intervention and others received other interventions.

MOD, Mode of Delivery; MP, medical professionalism; JSPE, Jefferson Scale of Physician Empathy; IRI, Interpersonal Reactivity Index.

### 3.3 Methodological quality of included reviews

The included systematic reviews were evaluated for methodological quality using AMSTAR 2 tool. The methodological quality across studies varied, with the majority scoring between low (*n* = 4) (25, 27, 28, 30) and critically low (*n* = 4) (23, 24, 26, 29); mainly due to lacking rigorous methodological designs, particularly in terms of bias assessment and reporting transparency (Table 3).

**Table 3:** Methodological quality assessment of reviews using A MeaSurement Tool to Assess systematic Reviews (AMSTAR) 2 scoring (22).

	AMSTAR items																
Author	1	2	3	4	5	6	7	8	9	10	11	12	13	14	15	16	Score
Franco et al., 2016 (23)	Y	PY	Y	PY	Y	Y	N	PY	Y	N	0	0	N	N	0	Y	Critically Low
Guraya et a., 2016 (24)	N	N	N	PY	Y	Y	N	Y	PY	Y	0	0	N	Y	0	Y	Critically Low
Ghosh et al., 2018 (25)	N	N	Y	PY	Y	Y	N	PY	N	Y	0	0	N	N	0	Y	Low
Lerchenfeldt et al., 2019 (26)	N	N	Y	PY	Y	Y	N	PY	PY	Y	0	0	Y	Y	0	Y	Critically Low
Rattani et al., 2021 (29)	N	N	N	PY	N	Y	N	PY	0	Y	0	0	Y	N	0	Y	Critically Low
Menezes et al., 2021 (28)	Y	PY	Y	PY	Y	Y	N	Y	Y	Y	0	0	Y	Y	0	Y	Low
Wang et al., 2022 (30)	Y	PY	Y	Y	Y	Y	N	N	Y	Y	0	0	Y	N	0	Y	Low
Leung et al., 2023 (27)	Y	Y	Y	PY	Y	Y	N	PY	Y	Y	0	0	Y	Y	0	Y	Low

Y = Yes, N=No, PY = Partial Yes.

### 3.4 Reported effectiveness of interventions on outcomes

Included reviews measured the effectiveness of educational interventions on two major topics: (i) MP as a whole (*n* = 4) (23, 24, 26, 29); (ii) specified empathy and compassion (*n* = 4) (25, 27, 28, 30); Leung et al. review have examined both topics and are discussed in both sections (27).

#### 3.4.1 Medical professionalism as a whole

Four systematic reviews reported the effectiveness of educational interventions of MP as a whole (23, 24, 26, 29). These educational interventions were delivered through various models and techniques. For instance, reflective practice (23, 24, 27), role modelling (24), audio-visual media (29), and collective peer feedback (23, 26). Due to the narrative nature of these reviews and the diversity of interventions, direct comparisons of effectiveness across studies were not feasible. However, certain trends emerged, with reflective practice and peer feedback standing out as particularly effective in fostering PB. Guraya et al.’ review systematically reported on the teaching strategies and their effectiveness in fostering MP and ability to analyse scenarios (i.e., identifying and analysing unprofessional behaviour) in UMS (24). The reviewers also identified multiple delivery modes of MP’s pillars, these include group-based discussion lectures, simulations, virtual reviews, preclinical teaching and experiential learning during clinical placement (24). Nevertheless, they concluded that the discussed heterogeneity suggests the absence of an evidence base to a consolidated approach in MP teaching (24).

Leung et al. review explored and discussed a structured reflective practice as a promising aspect to develop MP education and bridge the gap between theory and practice when delivered successfully (27). They concluded that future intervention should be further tested to validate successful provision of reflective practice (27).

Lerchenfedlt et al. assessed the effectiveness of ‘peer feedback’ during collective MP learning and denoted the likely value in improving MP and PB in UMS (26). The authors suggested (i) a standardized agreement on the definition of collective peer feedback and its assessment methods and (ii) added that future research could further explore this area, specifying assessments on the quality of these interventions on faculty and patients’ outcomes (26).

Rattani et al. (29) concluded that the use of trigger films in any MP teaching environment can improve engagement and fruitful discussions between UMS, especially in the current digital era. Trigger films, as explained by authors, characterised verbal conversations, non-verbal communication, reflective practice and inclusion of variety of different topics (29). The reviewers have suggested that trigger films should be relevant to scenarios experienced by medical students in clinical training (29).

Franco et al. (23) reported the use of portfolio as an effective tool in in reflective practice. Portfolios are reported to include an electronic ‘peer feedback’ discussion, defined in the review as either an assessment tool or a teaching approach, in which either have shown improvement in altruism as an attribute of MP (23). The authors discussed some degree of complexity that can contribute to failure of this approach, these include the timely process, lack of interest in some students and the use of scenarios irrelevant to practice (23). While these challenges exist, they suggested a framework to a support successful utility of portfolios that can adapted by researchers and educators (23).

#### 3.4.2 Empathy and compassion

As detailed in Table 4, four studies have systematically reported on the effectiveness of educational interventions on empathy and compassion (25, 27, 28, 30). The interventions varied widely, including reflective practice, virtual discussions, and group-based sessions, with mixed results depending on the context and delivery of the interventions. In three reviews, teaching techniques such as reflective practice, virtual discussions of donors, and in-person simulation, and group-based discussions have shown improvements in developing empathy and compassion in UMS (25, 27, 30). The majority of included interventions varied in outcomes and their assessment methods as detailed in Table 4. Authors have emphasized the importance of longitudinal exposure to the hidden curriculum by UMS at all levels of their education, from early exposure during their clinical placement stage (25, 27, 30).

**Table 4:** Effectiveness of MP educational interventions on outcomes.

Author, year	Franco et al. (23)	Guraya et al. (24)	Lerchenfeldt et al. (26)	Rattani et al. (29)
Medical professionalism as a whole				
Outcome	Perceived relevance and usefulness of portfolios. Impact on reflection and self-assessment Effectiveness in supporting the development of MP competencies	Not specified	Effectiveness of MP education, student learning and collaborative team dynamics.	Evaluate the perceived utility and application of using audiovisual media in MP
Assessment	Varied; questionnaires and/or thematic analysis	Not specified	Varied; quantitative questionnaires, narrative comment, focus groups, and interviews	Varied; Qualitative appraisal and thematic analysis
Key findings	Portfolios were well received by students and considered a valuable tool for teaching and learning to improve reflection and self-assessment. Portfolios were well-regarded for fostering PB with their versatility and focus on reflection being highlighted as significant strengths. However, challenges included were the artificiality of reflections, time-consuming processes, and student preferences for other teaching methods.	Lack of unified model for teaching MP Most common strands for teaching MP: role modelling, mentoring, hidden curriculum, reflective practice and effective communication.	Peer feedback in collaborative learning environments may be reliable for assessing MP and aids in PB development Mixed results regarding the impact of peer feedback on students’ collaborative team dynamic	Audiovisual media, particularly trigger films were considered effective and engaging tools to different teaching contexts and used as conversation catalyst through providing realistic scenarios.
Conclusion	The uses of portfolios in teaching MP is a promising strategy, particularly when reflection is effectively guided and assessed. The study proposed a framework for developing portfolios that foster professional behaviour.	There is no universally agreed model for teaching MP. The professional conduct of faculty role modelling and teaching core principles of reflective practice can encourage good MP practice through positive MP teaching.	Peer feedback is feasible and may be a useful method in MP education. However, training for both faculty and students is essential for effective implementation of this method.	Authors conclude that trigger films represent an effective and unique pedagogical strategy in supplementing current MP at undergraduate medical level
Review limitations	Small number of included interventions and their heterogeneity contribute to potential weakness of results.	Not reported	Language and publication biases Reporting bias of included studies; descriptive in nature thus limited drawing conclusion.	Selection bias of search strategy. Included interventions portrayed the educators perspective only.
Suggestions and implication	The review suggested that portfolios could be a powerful tool for teaching MP, but their success depends on careful implementation. Future research should focus on developing a standardised assessment method for portfolios and exploring the long-term impact of portfolios use on professional development.	International collaboration between academics to developing reflective practice and role modelling, targeted at improving patients care and professional excellence. More research is needed to explore the association between culture, versatility and gender in recruiting physician role-models and mentors	Further research is needed to standardize definitions for team dynamics for outcomes. There should be more focus on the quality of peer feedback on academic performance, institutional benefits and patients’ benefit.	Authors suggested that educators consider incorporating short films and audiovisual into their teaching to enhance student engagement and promote discussion. Trigger films could be a cost-effective, relevant, and adaptable method for enhancing MP education, particularly for digitally native medical students.
Empathy and compassion				
Author, year	Ghosh et al. (25)	Menezes et al. (28)	Wang et al. (30)	Leung et al. (27)
Outcome	Development of professionalism, empathy, and humanistic attributes among medical students.	Self-reported change in (1) knowledge, skills and attitude (2) Behaviour (3) Patient reported outcomes	Predictor of empathy and compassion	Empathy Professionalism
Assessment	Formal competency evaluation assessing professionalism through demonstration, commitment, behaviour, and core attributes.	Self-reporting questionnaire, behavioural assessment (e.g., standardised patient encounters, observed interactions) and patient reported outcomes measures (e.g., satisfaction surveys) Using Kirkpatrick model/MERSQUI scores/Jefferson scale of empathy.	Varied; Self-reporting questionnaires or qualitative interviews and focus group discussions or a combination of both.	Varied; quantitative measurement scales of empathy and qualitative analysis of reflective writing and discussion.
Key findings	Practices like showing donor interviews and involving donor families to: Humanize the dissection experience among medical students, fostering respect and compassion for the donor. Assess cultivating professionalism	Variety of teaching modalities (single/multiple modalities) were effective in improving empathy and compassion. There is a lack of continuity in teaching curricula of these topics.	Predictors of greater compassion included maturity, work and life experiences, openness to experience, perspective-taking, and positive role modelling. Conversely, negative attitudes, burnout, stress, and heavy workloads predicted lower compassion. The environment, including role models and the educational culture, significantly influences compassion in medical students.	Benefits of group reflective practice (when practiced voluntary): May help bridge the gap between theory and practice, foster collaboration, mitigate isolation through grasping biopsychological model of illness in the context of their patients Potentially preserve or enhance empathy in clinical placements if timing accurate—must be in clinical placements to ensure experiential learning
Conclusion	Incorporating humanistic practices in dissection curricula can significantly enhance MP among medical students and thus it is important to begin incorporating MP in the delivery of hidden curriculum.	Standard teaching using a blend of modalities should be introduced to emphasise compassion and empathy medical students.	Compassion in medical student is influenced by a wide range of personal, environmental, and clinical factors, with existing research primarily focusing on student-related factors.	Shows that group reflective practice may bring theory to life in clinical dilemmas, despite absence of studies directly examining wellbeing Early clinical exposure is helpful to medical students’ development of appropriate MP attitudes and can help foster socially responsive career choices.
Review limitation	Not reported	Language bias Limited reporting of a representative demographic content (gender, race, ethnicity) High risk of bias of included interventions and low confidence measure	Language and time bias. Heterogeneity in methods and outcomes measures of included interventions limited the study’s aim to: Highlighting the best practices for MP teaching. Reporting standardised curriculum.	Bias risk and applicability of results due to the variable quality of included interventions Lack of meta-analysis Strict search strategy Language bias
Suggestions and implications	Medical schools should adopt these practices in the overall anatomy education to yield more empathetic and compassionate physicians.	There is a need to develop a standardised curriculum and highlight best practices which is important for sustained programmes is essential to mitigate the risk of decline in empathy and compassion, rather than a single training activity. There is a need for more rigorous design and measurement studies in this area.	Future research should focus on exploring patient, environmental and clinical factors that influence compassion and to address biases in existing studies. There is also a need for interventions that target more identity formation factors like perspective-taking and mindfulness. Addressing educational environments that prioritize knowledge over compassionate care is critical. The review emphasized the need for more research into long-term impacts, particularly regarding environmental and clinical factors.	Evaluation of the impact of group reflective practice is needed to improve quality of curriculum and consistent methods of evaluation is also needed. Future research should directly examine the relationship between reflective practice and well being

MP, Medical professionalism; MERSQUI, Multidimensional Empathy Response Scale for Quality Improvement.

Menezes et al.’s (28) review identified various teaching programmes designed and resulted in improvement in empathy and compassion in UMS. While a comparison in the effectiveness between the different teaching programmes was not achievable, the review has highlighted the need for a longitudinal curriculum agenda that can potentially include a blended programme, focusing on interprofessional practice and professional identity formation (28).

Leung et al.’s (27) review—the most recent included review—on the other hand, identified collective reflective practice in UMS as a promising approach to enhance and retain empathy and compassion, when exercised voluntarily. Similar to the majority of included reviews in our study, the authors included interventions provided to students at both pre and during clinical placement stage. Nonetheless, Leung et al. (27) highlighted the importance of the consistent learning during clinical placements and suggested that future research should examine the quality of MP teaching evaluation methods to maintain PB. The reviewer also assessed the effectiveness of wellbeing and reported that the lack of literature to provide evidence suggests that future research can explore the influence of collective reflective practice on the wellbeing of UMS (27).

Wang et al. (24) reported on the predictors of empathy and compassion, focusing on personal factors (e.g., cultural background, education level) and environmental factors (e.g., the educational culture and role modelling). While personal factors had inconsistent effects, environmental factors, particularly positive role modelling, were found to have a critical impact on developing empathy and compassion in medical students (30).

Ghosh et al. (25) focused on examining formation of MP and empathy in UMS using recorded video interviews of donors and meeting the donors’ family members in dissection anatomy courses. The review reported a noted improvement in empathy and compassion as a result of the hidden curriculum exposure using patient factors and authors discussed the importance of this approach on MP education and development of professionalism in practice (25).

### 3.5 Sustainability of medical professionalism education

Six included reviews have reported details on the potential sustainability of MP education from included interventions and was dependent of specific implementation strategies used in each study. Leung et al. and Ghosh et al. have reported that collective reflective practice and digital clips of donors in dissection anatomy can foster PB and MP traits (25, 27). The authors have discussed the importance of linear exposure to hidden curriculum not only during the preclinical stage but also during their clinical training to support experiential learning and prolong positive development (25, 27). Ghosh et al. concluded on the promising impact of interventions included in preserving empathy and compassion (25).

Menezes et al. have emphasized the importance of the sustainability of MP teaching and learning provided to UMS as it can potentially support professionalism in practice (28). Franco et al. discussed challenges to achieving effective results and suggested that evaluation of the impact of teaching tools can support their term effects (23). Wang et al. informed the limited literature on the long-term effect of factors that influence PB in medical students and suggested further investigation from future research in this area (30). Lerchenfeldt et al. review did not report on long-term effect of included MP educational interventions, but concluded that future studies should further develop interventions and study benefits to professionalism in practice (26).

Overall, there is limited data on the long-term sustainability of professionalism education, with few studies evaluating the lasting impact of these interventions. This highlights a gap regarding the durability of professionalism education outcomes.

## 4 Discussion

This review included an overview of SRs of interventions published in the last three decades including over 100 thousand UMS. These SRs included various study types that narratively assessed interventions of a wide range of teaching modalities aimed at improving MP as a whole and empathy and compassion. While some SR reported a degree of success in teaching techniques, there remains limited evidence on a standardized approach to MP education in UMS. All included systematic reviews presented a low-quality score and limited results were identified to provide evidence on the long-term impact of MP educational interventions.

While MP education is vital to UMS, published interventions in this area to date are limited in evaluating and developing MP in research (31). The limitation can stem from challenges given the inconsistent definition of MP education’s and its uniformly across different contexts (32). In other words, aspects of MP, their interpretation and application can vary widely among individuals and institutions (33). This review adds to the body of evidence on significant challenge that is the lack of standardized, objective tools for measuring professionalism (2). Passi et al. explain that the nature of MP education in different contexts are often prone to bias and subjectivity, complicating efforts to produce developing curriculum on professionalism (18). Moreover, Mueller et al. adds the complications in designing a universal framework due to personal and cultural factors (34). Similar interpretations can be drawn from our review to reflect on the importance of the cultural sensitivity influence on the formation of an effective curriculum to developing PB in UMS. These complexities hinder the creation of a clear, evidence-based framework for research on professionalism. Thus, this review suggests that establishing the taxonomy of MP definition and learning outcomes is essential to support the development of a standardised/innovative assessment of MP educational syllabus, accommodating its dynamic and context-dependent nature. This suggestion is similar to a systematic review conducted by Al Rumayyan et al. on the differences between MP frameworks across multiple geographic regions (35).

Included SRs includes UMS at the preclinical and clinical stages of their educations. While results did not allow our review to draw comparisons between the two stages, it is important to acknowledge the growing research reporting the fundamental impact on their PB. On one hand, evolving interventions are focused on students on their preclinical stage for the benefits of developing PB and preparation for unprofessional dilemmas through critical learning and reflections (15, 36, 37). On the other hand, other interventions aim at targeting students in their later stage with focus on experiential learning (38, 39). These interventions are based on a theoretical perspective of behaviour and learning (40, 41) given their significance in explaining what works and does not work in an intervention. Additionally, the use of theory benefits the feedback loop in learning and bridging gaps between theory and practice (38, 41). This review suggests that future research should focus on a longitudinal curriculum design for different stages of learning that is based on a theoretical perspective and is needed to tackle gaps in professionalism in practice.

To date, there is limited evidence on the sustainability of MP educational interventions. Carr et al. suggests the complexity in designing interventions aimed at exploring long term impact on outcomes (42). This review could not report of barriers and facilitators to studying the long-term impact of educational interventions included within SRs. Thus, the review highlights the ambiguity in literature reporting in this area. Furthermore, a wealth of research had been conducted towards the importance of extended learning of professionalism at the postgraduate level to further sustain PB and patient safety (43–45). This has emerged from the reported evidence of the declining in professionalism traits as part of the professional identity formation in residents and healthcare professionals (46). Ultimately, fostering professionalism at the postgraduate level is crucial for ensuring that healthcare professionals are equipped not only with technical expertise but also the ethical and interpersonal skills needed for professional and humanistic patient care. More research, particularly longitudinal randomised controlled trials are needed to understand the evolution of professionalism education at the undergraduate level and if postgraduate education is essential for the sustainability of PB.

### 4.1 Limitations

The potential always exists in reviewing extensive literature that important studies may have been missed either during the screening of SR published in English language and/or using systematic reviews as the unit of analysis not the original interventions. Secondly, while the search strategy was developed with a team of experts and subject librarian in the Royal College of Surgeons in Ireland, some keywords used to describe either MP or educational interventions, given the discussed inconsistent definitions of MP, could have omitted. Lastly, it is important to acknowledge that all included reviews exhibit a low methodological quality which consequently have a minor impact on conclusions drawn from this umbrella review.

## 5 Conclusion

The majority of included reviews have reported a successful improvement in various aspects of MP (i.e., MP as a whole, empathy and compassion) through teaching and exposure to hidden curriculum in UMS. A gap is still present in reporting the sustainable effect on professionalism traits in UMS and on suggesting a standardised approach to professionalism teaching and improvement in professionalism in practice. This review suggests that (1) future research should be towards a systematic review of methodological quality to support rigour interpretation; and (2) more educational interventions are needed in this area, with the focus on teaching methods in multicultural context to support professional identity formation and precursors of PB.

## Data Availability

The original contributions presented in the study are included in the article/supplementary material, further inquiries can be directed to the corresponding author.
